# Analysis of Resin-Based Dental Materials’ Composition Depending on Their Clinical Applications

**DOI:** 10.3390/polym16081022

**Published:** 2024-04-09

**Authors:** Claire-Adeline Dantagnan, Sylvie Babajko, Ali Nassif, Sophia Houari, Katia Jedeon, Philippe François, Elisabeth Dursun, Jean-Pierre Attal, Julia Bosco

**Affiliations:** 1 Innovative Dental Materials and Interfaces Research Unit, Faculty of Dentistry, Université Paris Cité, 1 rue Maurice Arnoux, 92120 Montrouge, France; cl.dantagnan@gmail.com (C.-A.D.); philo.françois@gmail.com (P.F.); elisabethdursun@gmail.com (E.D.); julia.bosco@u-paris.fr (J.B.); 2 Pitié-Salpêtrière Hospital, 47-83 Boulevard de l’Hôpital, 75013 Paris, France; ali.nassif@u-paris.fr (A.N.); houarisophia@hotmail.fr (S.H.); 3 Biomedical Research in Odontology, Faculty of Dentistry, Université Paris Cité, 1 rue Maurice Arnoux, 92120 Montrouge, France; sylvie.babajko@inserm.fr (S.B.); katia.jedeon@u-paris.fr (K.J.); 4 Rothschild Hospital, 5 rue Santerre, 75012 Paris, France; 5 Bretonneau Hospital, 23 rue Joseph de Maistre, 75018 Paris, France; 6 Henri Mondor Hospital, 1 rue Gustave Eiffel, 94000 Créteil, France; 7 Charles Foix Hospital, 7 Avenue de la République, 94200 Ivry sur Seine, France

**Keywords:** bisphenol A, endocrine disruptors, dentistry, resins

## Abstract

The objective of this study was to detail the monomer composition of resin-based dental materials sold in the market in 2023 and to evaluate the proportion of bisphenol A (BPA)-derivatives in relation to their applications. A search on manufacturers’ websites was performed to reference resin-based dental materials currently on the European market (including the European Union (EU) and United Kingdom (UK). Their monomer composition was determined using material-safety data sheets and was completed by a search on the PubMed database. Among the 543 material compositions exploitable, 382 (70.3%) contained BPA derivatives. Among them, 56.2% contained BisGMA and 28% BisEMA, the most frequently reported. A total of 59 monomers, of which six were BPA derivatives, were found. In total, 309 materials (56.9%) contained UDMA and 292 (53.8%) TEGDMA. Less than one third of materials identified contained no BPA derivatives. These proportions vary a lot depending on their applications, with materials dedicated to the dental care of young populations containing the highest proportions of BPA-derivative monomers. The long-term effects on human health of the different monomers identified including BPA-derivative monomers is a source of concern. For children and pregnant or lactating women arises the question of whether to take a precautionary principle and avoid the use of resin-based dental materials likely to release BPA by opting for alternative materials.

## 1. Introduction

Nowadays, resin-based dental materials are commonly used in multiple fields of dentistry with a wide range of applications: restorative dentistry with resin-based composite materials and their associated adhesive systems [1,2] or resin-modified glass ionomer cements [3] to restore decayed, worn, or traumatized teeth, pediatric dentistry with pit and fissure sealants [4], orthodontics with adhesives for brackets or fixed retainer bonding [5], and prosthetic dentistry with luting cements and composites [6].

Resin-based dental materials are generally composed of an organic matrix made of oligomers and monomers with inorganic or organic filler particles linked to the matrix through a silane coupling agent [1]. The monomers most frequently used are methacrylates. Among methacrylates, Bisphenol A glycidyl methacrylate (BisGMA) and other Bisphenol A (BPA)-derivative monomers are the most employed because of their properties such as flexural strength, volumetric shrinkage, water sorption, solubility, and viscosity [1,2]. Other methacrylates frequently used, alone or in association with BisGMA, are not bisphenol A-derived, such as Urethane dimethacrylate (UDMA), Triethylene glycol dimethacrylate (TEGDMA), or 2-hydroxyethyl methacrylate (HEMA) [1,2]. Moreover, recently, new types of resin matrix have been introduced such as ormocers and siloranes [1,2].

In recent years, the increasing presence of resin-based dental materials in oral cavity has raised questions about biocompatibility and safety of resin-matrix components [1,2]. Resin-based dental materials are subjected to numerous constraints that may be physical–chemical (thermal variations), mechanical (abrasion linked to oral hygiene measures and functions or parafunctions), chemical (corrosion caused by food and drinks, acid attacks, pH variations or hydrolysis), or even bacteriological (bacterial enzymatic attacks) [7,8,9,10]. These constraints and the context of the oral environment inevitably lead to their degradation and consequently the release of all or part of their components [8,9,10]. In addition, during the polymerization reaction of resin-based materials, monomers are converted into polymers mostly initiated by the light-curing of the material [11]. However, this conversion reaction is never complete, with residual monomers and the degree of conversion ranging, for example, between 50 and 80% for composite resins, allowing for the leaching of non-polymerized monomers [11]. High concentrations of substances released can lead to cytotoxic effects [10,12], genotoxic effects [12], or allergic effects [13]. However, the quantity of substances released would be too low to induce systemic toxicity [10,12,13].

Among components likely to be released, Bisphenol A, an organic compound listed as an endocrine disruptor by the European commission [14], retains particular attention because it may alter patients’ health at very low doses [15]. This molecule is commonly used in the industrial production of polycarbonate plastics and epoxy resins [14]. In dentistry, Bisphenol A is never found in a pure state in resin-based materials, but it can be released in the oral cavity [16] because it is the precursor of certain monomers used in their composition such as BisGMA, Bisphenol A dimethacrylate (BisDMA), Ethoxylated bisphenol A glycol dimethacrylate (BisEMA), Polycarbonate-modified BisGMA (PC BisGMA), and 2,2-bis[(4-methacryloxy polyethoxy)phenyl]propane (BisMPEPP) [16,17,18,19,20,21]. BPA may be detected in the plasma and saliva of patients treated with resins leaching monomer-derived residues [16,17,18,19,20,21].

The Bisphenol A found from resin-based dental materials can come either from impurities in the synthesis of BPA-derivative monomers or from their degradation over time [16,17,18,19,20,21]. However, among BPA-derivatives, only the cleavage of BisDMA by salivary esterases can directly form BPA [17,18]. For this reason, BisDMA is almost no longer used in dental resin materials.

Numerous experimental and epidemiological studies established the causal link between exposures to endocrine-disrupting chemicals, including BPA, and the development of certain pathologies [14,22] such as male and female infertility, early puberty in girls, breast, testicular, or prostate cancer, metabolic disorders (type-II diabetes, obesity, etc.), neurodevelopmental damage and behavioral disorders, or even enamel hypomineralising pathologies [23]. Infants, young children, teenagers during puberty, and pregnant and lactating women are the most sensitive to exposures to this substance [14]. Despite BPA contamination may occur through dermal, respiratory, or placental routes, the main contamination occurs through the oral route with alimentation and drinks containing this substance [14]. That’s why the European Commission has banned this molecule from the manufacturing of baby bottles since 2011, from plastic bottles and packaging containing foods for children under 3 years old since February 2018, and from thermal paper since January 2020 [14]. In 2023, the European Food Safety Authorities (EFSA) reduced the Tolerable Daily Intake (TDI) from 4 µg/kg/day to 0.2 ng/kg/day (20,000 times less than the last TDI) because of possible BPA low-dose effects [14].

To date, there is no study focused on the monomer composition of all categories of resin-based dental materials currently on the market that would help to evaluate the appropriate use of these materials. The available studies are mainly focused on only one category of materials, mainly composite resins or adhesive systems [1,20]. The goal of this study was first to detail the monomer composition of resin-based dental materials sold in the European market in 2023. Due to multiple concerns about BPA, the second aim was to evaluate the percentage of materials with BPA derivatives in their manufacturing in relation to their applications to inform practitioners about their possible risks for specific populations and to formulate recommendations for patient care.

## 2. Materials and Methods

### 2.1. Materials Studied

To reference an exhaustive list of resin-based dental materials sold in the European market (including the EU and UK), a search was conducted on the manufacturers’ websites. The following categories of dental materials were selected: three types of composite resins (restorative, orthodontic, and core build-up), two types of adhesive systems (restorative and orthodontic), sealants, restorative resin-modified glass ionomer cements, and luting resin-modified glass ionomer cements and composites.

### 2.2. Search Strategy

Next, for all the materials found, the chemical composition was searched on the material-safety data sheet (MSDS), when available, to reference all monomers contained in material composition. When the information was not available on the MSDS or on the manufacturer’s website, a complementary search was conducted on the PubMed database (National Library of Medicine) to identify studies with information on monomers’ composition of selected materials.

### 2.3. Data Analysis

All results were recorded and analyzed by using Microsoft Excel 2016 software (Microsoft, Redmond, WA, USA). Percentages were carried out to analyze the results for each material category. Then, the results were summarized in tables.

## 3. Results

### 3.1. Materials Identified and Source of Information

In 2023, a total of 743 resin-based dental materials were identified from 52 companies, respectively:

- A total of 305 restorative composite resins;
- A total of 49 core build-up composite resins;
- A total of 66 orthodontic composite resins;
- A total of 142 restorative adhesive systems;
- A total of 33 orthodontic adhesive systems;
- A total of 32 sealants;
- A total of 16 restorative resin-modified glass ionomer cements;
- A total of 100 luting resin-modified glass ionomer cements and composites.

Manufacturers and number of materials initially listed were presented in Supplementary Materials.

**Figure 1:**
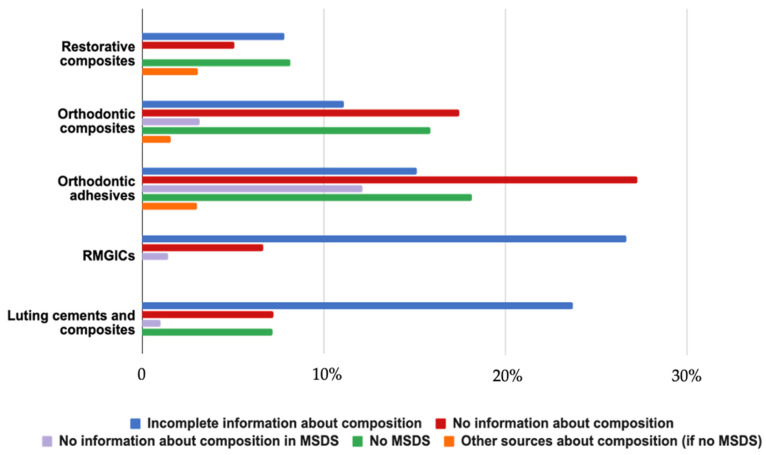
Information about chemical composition in all categories of materials.

Among all resin-based dental materials identified, 141 (19%) had insufficient information about their monomer composition, and 59 (7.9%) materials identified had no information about their monomer composition (Figure 1). As a result, the percentages were calculated considering the 543 products from 44 companies with known compositions (Table 1).

Materials were excluded when the information was incomplete, and the terms mentioned in the MSDS are reported in Table 2. The most frequently mentioned terms were “methacrylates”, “blend of multifunctional methacrylates”, “hydrophobic aromatic dimethacrylate”, “hydrophobic aliphatic dimethacrylate”, “uncured methacrylate ester monomers”, “acid adhesive monomer”, “hydrophilic aliphatic methacrylate”, and “acidic monomer” (Table 2).

### 3.2. Monomers Identified

**Figure 2:**
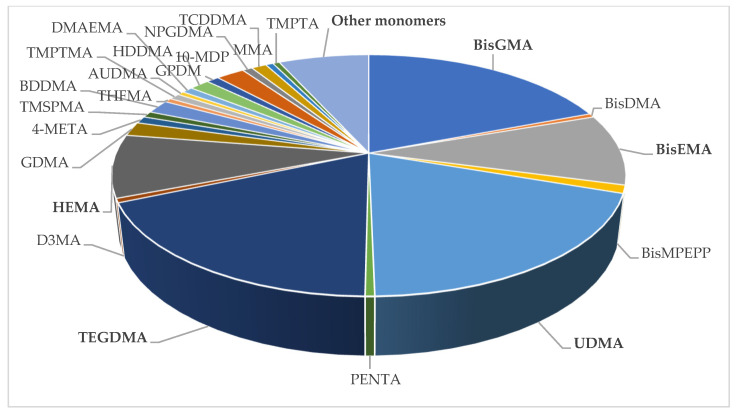
Global repartition of the different monomers identified.

**Figure 3:**
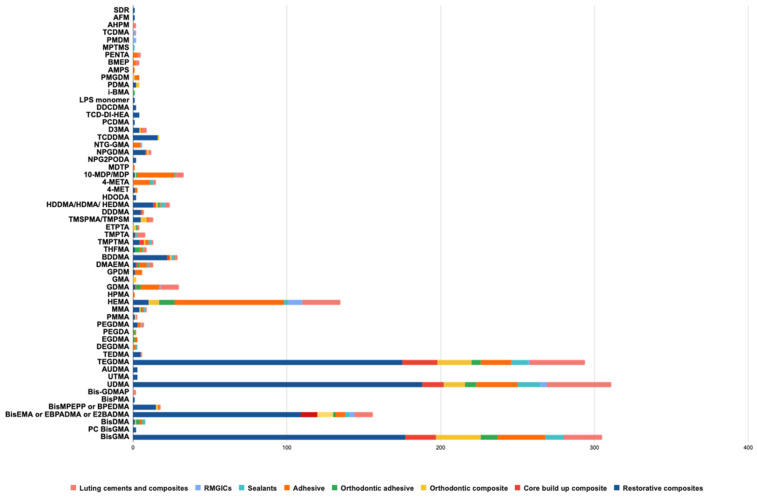
Repartition of the different monomers identified in all categories of materials (number).

**Figure 4:**
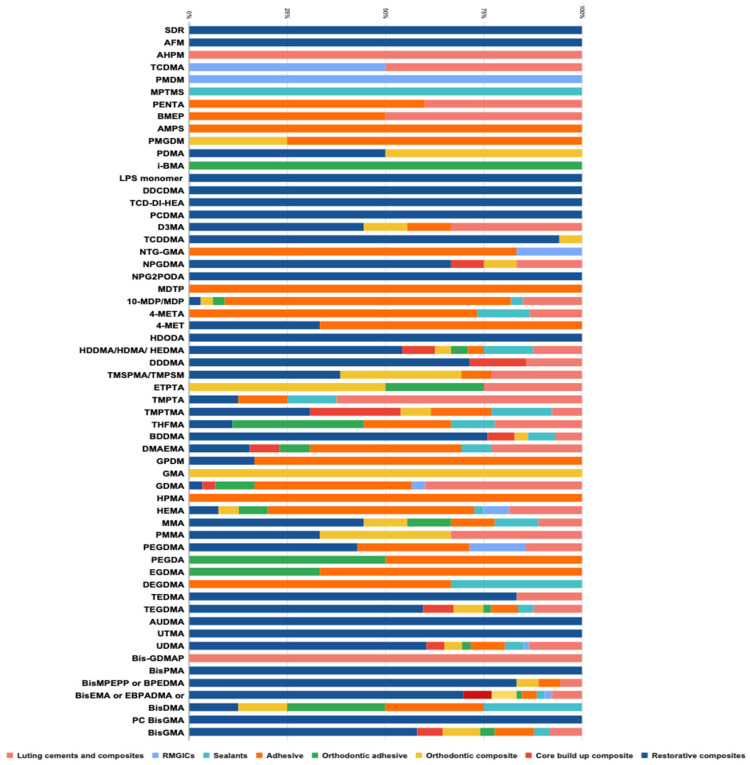
Repartition of all monomers identified in all categories of materials (percentages).

In total, 59 monomers were found in the chemical compositions of all materials, and 6 were BPA derivatives (Table 3). One composite resin contained silorane and five composite resins contained an ormocer resin matrix. The global repartition of the identified monomers in all materials screened is presented in Figure 2. The repartition of the identified monomers in all categories of materials is presented in Figure 3 and Figure 4.

Among all materials included in the final analysis (543 materials), 382 materials (70.3%) contained BPA derivatives. Among them, 305 (56.2%) contained BisGMA, 152 (28%) contained BisEMA, 17 (3.1%) contained BisMPEPP, 8 (1.5%) contained BisDMA, 3 (0.6%) contained BisPMA, and 2 (0.4%) contained PC BisGMA.

In total, 161 materials (29.7%) contained no BPA derivatives. Among all with no BPA-derivative monomers identified, UDMA, TEGDMA, and HEMA were the most common in all resin-based materials categories. In total, 309 materials (56.9%) contained UDMA, 292 (53.8%) contained TEGDMA, and 134 (24.7%) contained HEMA. Twenty-one materials (3.9%) contained no BPA derivatives and no UDMA, TEGDMA, and HEMA (Table 4). The highest proportion of BPA-derivative materials was found in composites (78.7 to 83.8%). Some materials still contained BisDMA, such as 10.5% of orthodontic adhesives and 8.3% of sealants.

## 4. Discussion

In this study, an exhaustive list of 743 resin-based materials currently marketed from 52 companies was drawn up. Among them, 543 are provided with a complete composition list and were included in the study for the final analysis.

### 4.1. Source of Information

Patients may ask their practitioner about the nature and safety of materials placed in their oral cavity especially concerning possible BPA derivatives. Practitioners have a duty to inform and protect their patients. This means they must know the chemical compositions of all the materials used for traceability requirements.

In this study, the composition of each category of resin-based dental materials identified was well established. Among the 743 materials initially found, 141 (19%) had insufficient information about their monomer composition and only 59 (7.9%) had no information about their monomer composition. It was thus possible to work on 543 products. In fact, sometimes manufacturers are reluctant to reveal all the components in their products due to commercial reasons and trade secrets. Currently, there is no obligation to communicate the exact composition of materials, unlike what it is required for drugs. The material safety data sheet (MSDS) of a product should give information on all its components with a proportion above 1% (REACH Regulation (EC) 1907:2006 in the European Union, OSHA Hazard Communication Regulations 1910.1200g8 for the United States). However, this study showed that several MSDS forms indicate only part of the composition, mentioning only the family of molecules like “methacrylates”, “hydrophobic aliphatic dimethacrylate”, or “hydrophobic aromatic dimethacrylate”. In addition, some components may have undesirable long-term effects on health despite their presence at low-doses when released chronically during decades. That is why it is important that manufacturers provide the complete and precise list of potentially active substances (even if present < 1%).

### 4.2. Concerns about Bisphenol A-Derivative Monomers

Our study indicates that most dental materials (70.3%) sold in 2023 still contain BPA-derivative monomers. It is generally admitted that this substance leached from dental material is not likely to pose a threat to human health [20,21], and the situation should be analyzed carefully with more detail. In fact, the most used dental materials, especially for the care of children and teenagers, such as restorative composites, orthodontic composites, orthodontic adhesives and sealants, are the most susceptible to containing BPA-derivative monomers, with values of 83.8%, 78.7%, 63.2%, and 66.7% for them, respectively. BisGMA was the most-often reported BPA derivative (56.2%) except for resin-modified glass ionomer cements. BisEMA is the second BPA derivative most frequently present in 28.1% of dental materials, in all categories of materials except adhesives.

The release of BPA from resin-based dental materials is described in the literature both in vitro in organic solvents or artificial saliva and clinically in saliva or urine [16,17,18,19,20,21,24,25,26,27]. The data in the literature show a great heterogeneity for this substance’s levels, which vary from one study to another [16,17,18,19,20,21,24,25,26,27]. In fact, BPA levels depend on analysis techniques, extraction solutions, fixed detection thresholds, or other experimental conditions, which make studies difficult to compare [15,16,24]. For example, with the GC/MS (Gas chromatography/Mass Spectrometry) technique, the application of heat can overestimate the concentrations of BPA released because it accelerates the process of degradation of BPA-derivative monomers into BPA [17,18,26]. Also, measurements of BPA levels are performed at different times after starting the in vitro degradation procedure, with a maximum elution found after 24 h [16,17,18,19,20,21,24,27].

The reported quantities of BPA measured in patients’ saliva are generally higher than those released in vitro in artificial saliva or buffers (around 10 to 100 times higher) [17,26]. As a reminder, this substance’s levels in patients’ saliva reported in the first paper of Olea and co-workers were from 3 to 30 mg/mL [16], whereas a recent study evaluating the BPA release immediately after composite resin filling in adults found much lower levels of this molecule, with a mean level at 0.11 ng/mL [25]. Like in vitro studies, clinical studies are difficult to compare. Studies that do not present an acidification step in their saliva recovery procedure can overestimate the BPA found due to the process of degradation of BPA-derivative monomers into BPA, which is accelerated without this acidification step [25]. Furthermore, individual factors can influence the amount of this substance in saliva, including patients’ lifestyle, the respect of the instructions before saliva-sample collection, the metabolism of molecules by the salivary enzymes, and the volume of resin material used [25].

The BPA leached from resin-based dental materials can come either from impurities in the synthesis of monomers in their chemical composition or from the degradation of BPA-derivative monomers over time (only BisDMA cleavage by salivary esterases can release pure Bisphenol A) [16,17,18,19,20,21]. BisDMA is relatively rare in resin-based dental materials, which is confirmed by our study (1.5% of all dental materials). However, when considering the situation more carefully, its presence was still found in 10.5% of orthodontic adhesives and 8.3% of sealants. For example, a study shows a cumulative BPA level in saliva of 0.09 ng during the first 24 h from four dental sealants (four sealants corresponding to 32 ng of resin used) [27]. Although this substance’s levels reported in the literature are generally below the tolerable daily intake (up to 2 ng for 6-year-old children weighing 20 kg according to the recently TDI set by EFSA), these data are surprising because they concern young people, a population more susceptible to the long-term effects of environmental toxicants even at low doses [14,15,22].

Despite BisDMA is generally admitted as the sole BPA-derivative monomer able to release BPA, De Nys et al. showed conversion rates of BPA in artificial saliva at 0.0003% for BisGMA and at 0.0017% for one type of BisEMA [18]. These values could seem relatively low, but these two monomers are the most widespread in dental materials (for BisGMA, around 10–25% of its weight in the resin matrix of certain restorative composites); they thus represent a non-negligible amount of BPA possibly released.

The major health impacts and concerns of BPA are linked to its endocrine-disrupting activity after years of chronic exposure [14,22]. Certain periods of life should be considered with precaution: pregnancy with fetal organ development, the newborn stage, due to tissue immaturity, and adolescents during puberty, with the maturation of sexual organs (around 12–15 years old for boys and 10–12 years old for girls) [14,22]. However, due to transgenerational BPA activity, all individuals capable of procreating should be considered carefully, which enlarges the period of critical time for this molecule exposure [28]. In addition to the window time of exposure, the dose of exposure must also be considered, as BPA may have greater effects at low doses than high doses without a threshold dose, contrary to the classic pattern encountered in toxicology [29]. Recently, the European Food Safety Authority decided to reduce the Tolerable Daily Intake allowed to 0.2 ng/kg/day, which is lower than the BPA levels detected in saliva after resin placement in the oral cavity [14]. This point should be considered for the recommendation of materials completely devoid of BPA-derivative monomers placed for years in the oral cavity. The continual chronic leaching of monomers able to be degraded into BPA, even at very low dose, may have long-term side effects on patients’ health [22,23,28]. In addition, as this molecule sublingual passage into circulation is possible, it may be immediately active on target tissues [30].

### 4.3. Other Types of Monomers

In total, 161 of the 543 resin-based dental materials analyzed in this study (29.7%) contained no BPA-derivative monomers. Bisphenol A is not the only potentially toxic component in resin-based dental materials; other monomers could be toxic. The release of UDMA, TEGDMA, and HEMA is often reported in the literature, whether in vitro in organic solvents or artificial saliva or clinically in saliva [11,17,26,31]. Among the 53 monomers that are not BPA derivatives, three are mainly found in the chemical composition of the different categories of materials studied: UDMA, TEGDMA, and HEMA.

In the context of resin-based materials, it is recognized that the presence of unpolymerized monomers can cause toxic biological effects such as cytotoxicity, estrogenicity, genotoxicity, or allergic reactions [10,12]. These effects are rarely immediate. Furthermore, the higher the degree of polymerization is, the less toxic the biological effects observed would be [10,12].

Some authors demonstrated adverse effects depending on the presence of TEGDMA, HEMA, or UDMA [32,33,34,35,36,37]. UDMA is a monomer commonly added in dental resin-based dental materials to enhance their viscosity and is considered an alternative to BisGMA in these materials [1,2]. A recent meta-analysis reported UDMA toxicity on fibroblasts or mesenchymal cells just below BPA but higher than TEGDMA and HEMA [32]. Resin-matrix cements cause a cytotoxic reaction when in contact with fibroblasts or mesenchymal cells due to the release of monomers from the polymeric matrix. The amounts of monomers released from the resin matrix and their cytotoxicity depend on the polymerization parameters [32]. UDMA was found in all categories of materials screened in this study and was the most widespread monomer in sealants and luting cements and composites. UDMA presents some cell toxicity and genotoxic effects for some cell types (pulp cells, human gingival fibroblasts or even macrophages) [32,33,34]. These effects occur even at very low UDMA concentrations, suggesting low-dose effects of this monomer on health comparable to BPA, as earlier discussed [33]. However, often, UDMA used in resin-based materials is in a modified form, as mentioned in certain MSDSs (Aromatic Urethane Dimethacrylate (AUDMA), urethane methacrylate oligomer, or UDMA-modified). Modified UDMA should be further investigated for their cellular activities, as no data are available to date concerning their possible low-dose and long-term effects.

TEGDMA is a low-molecular-mass monomer often added into resin-based dental materials’ matrixes to reduce the viscosity of the mixture [1,2]. In this study, it was present in all categories of materials except RMGICs and was the second monomer most frequently found in orthodontic composite resins and luting cements and composites. It has been reported that TEGDMA presents cytotoxic, genotoxic, and estrogenic effects for different cell types such as pulp cells, human gingival fibroblasts, and monocytes but at a lower level than UDMA [34,35]. TEGDMA, contrary to UDMA, is also able to activate estrogen receptor alpha at low doses, like for Bisphenol A and its derivatives [36].

HEMA is a low-molecular-mass monomer with a hydrophilic character. It is frequently added to the resin matrix of adhesive systems and luting cements and composites [1,2]. HEMA is the monomer used in the resin matrixes of RMGICs [3]. Accordingly, HEMA was also found the most frequently in adhesive systems and RMGICs. HEMA may present some cell toxicity and genotoxic effects but much lower than those of BPA, BPA derivatives, UDMA, or TEGDMA [37].

Finally, the combination of certain monomers could increase the cytotoxic and genotoxic effects observed, as has been shown in the case of the combination of TEGDMA with UDMA often found in resin-based materials [34]. However, concerning monomers’ toxicity, there are only few clinical studies and studies, which are mainly carried out in vitro. This does not fully reflect the conditions of the oral environment, particularly the role of saliva. 

### 4.4. Limitations of the Study and Perspectives

One limitation of this study was that the search conducted was only based on MSDS pages. It would have been interesting to add to this research by contacting the different manufacturers identified to cross-reference the composition information in MSDSs with their responses. This study focuses on the monomer composition of resin-based dental materials in relation to their potential toxicity. Other components present in the chemical composition of these materials like polymerization initiators, stabilizers, viscosity reducers, etc., have also demonstrated a potential toxicity in the literature [38,39]. Future research could focus on the distribution of these other components among resin-based dental materials’ composition in relation to their toxicity.

### 4.5. Clinical Recommendations

All studies on the activity of monomers released from dental materials have led to proposing a limited exposure to unpolymerized resin-based dental materials and to selecting materials without BPA derivatives for children and teenagers. The same precautions must be taken for pregnant and lactating women. Some clinical procedures could be applied to minimize the release of unpolymerized monomers [17,18,19,20,21,40,41,42]: rubber dam use for restoration making, using a curing lamp with sufficient power (>1000 mW/cm^2^), bringing the fiber of the curing lamp closer to the material to be cured, and prolonging curing time or, in the case of restoration making, adding a second curing step after covering the restoration with a glycerin film. Unpolymerized monomers are present on the surface of the material because of the inhibition of polymerization induced by oxygen [40,41]. It was also demonstrated that brushing the restoration surface with pumice or water/air spray eliminated most residual monomers [41,42]. A gargle with warm water 30 s after orthodontic bonding [20] or restoration bonding [41] could also reduce the level of residual monomers. Finally, using indirect or CAD-CAM resin materials for restorations could also minimize the monomer release with a maximum degree of conversion for these types of materials [42].

Despite the part of the overall BPA exposure dose coming from oral intake linked to dental materials, is difficult to evaluate precisely; the contribution of dental materials to overall BPA contamination is not negligible. When considering the possible cocktail of effects, in individuals chronically exposed to a multitude of toxic substances, these substances, combined with each other, may have greater undesirable effects for the body, increasing the concern about dental materials containing BPA-derivative monomers [43]. According to the last TDI for BPA established recently by EFSA, this substance is formerly banned from the environment of Europeans [14]. To limit the exposure of patients to components likely to release BPA, some manufacturers developed alternative substitutes of this molecule, as bisphenol S or bisphenol F are also classified as endocrine disruptors based on studies showing similar effects to BPA [44]. An alternative was resin-based dental materials without BPA derivatives but still containing other types of monomers such as UDMA or TEGDMA [17,18]. Another alternative could be using materials without resin for restoration making or orthodontic bonding, such as high-viscosity glass ionomers and ceramic (for restorations).

## 5. Conclusions

Despite how it may generally be admitted that resin-based dental materials are of no concern for human health, it is necessary to carefully analyze their composition to evaluate their hazards and risks for specific populations to propose recommendations for patient care.

This study has established an exhaustive list of 543 resin-based dental materials from 44 companies. Among their chemical composition, 59 monomers were found, with 6 being BPA derivatives. More than 70% of materials, including composite resins and adhesive systems for restorative dentistry and orthodontics, sealants, luting cements, and composites and RMGICs, contain BPA-derivative monomers. More importantly, some materials mostly used for young populations such as composite resins and adhesives for restorative dentistry and orthodontics still contain BisDMA, which is able to release Bisphenol A. The long-term effects on human health of the different monomers identified, BPA in particular, are now well established. That is why, considering the possible health impact of its derived monomers, whatever their levels of release in the patient body, practitioners should opt for alternative materials that do not contain any BPA-derived monomers and, at least, materials provided with an MSDS listing their exact chemical compositions, like what is done for drugs.

This precautionary recommendation would be the responsibility of dentists and of competent health authorities.

## Figures and Tables

**Table 1:** Manufacturers and number of materials identified for final analysis.

Manufacturer	Restorative Composite Resins	Core Build-Up Composite Resins	Orthodontic Composite Resins	Restorative Adhesive Systems	Orthodontic Adhesive Systems	Sealants	Restorative RMGICs	Luting Cements and Composites
Apol	5	/	/	1	/	/	/	/
American orthodontics	/	/	1	/	1	/	/	/
Bisico	12	3	/	6	/	1	/	4
BJM	/	/	2	/	1	/	/	/
Cavex	3	/	/	2	/	/	/	/
Coltene	11	1	/	5	/	/	/	3
Cosmedent	8	1	/	/	/	2	/	2
CyberTech	2	1	/	/	/	/	/	/
Dentaurum	/	/	3	/	/	/	/	/
DenMat	2	1	/	6	/	1	2	/
Dental Technologies	5	2	2	4	1	2	/	2
Dentsply	14	2	/	4	/	1	/	3
DMG	6	2	/	4	/	/	/	2
Exotec	2	/	/	/	/	/	/	/
FGM	5	1	/	/	/	/	/	2
GC	20	1	2	3	/	/	2	10
Gestenco	/	/	1	/	/	/	/	/
Henry Schein	7	/	/	2	/	1	/	1
Itena	2	1	/	2	/	1	/	3
Ivoclar-Vivadent	15	2	1	7	/	4	/	4
Jeneric Pentron	5	2	/	2	/	/	/	/
Kerr	11	/	/	5	/	/	/	4
Kettenbach Dental	2	/	/	/	/	/	/	1
Kulzer	15	/	/	3	/	/	/	/
Kuraray	6	3	/	/	/	1	/	1
3M	12	/	6	8	2	2	5	3
Micerium	5	/	/	/	/	1	/	4
Ormco	/	/	4	/	1	/	/	/
Ortho Technology	/	/	3	/	/	/	/	/
Parkell	2	2	/	2	/	/	/	2
Reliance	/	/	13	/	10	/	/	/
RMO	/	/	3	/	1	/	/	/
R and S	3	/	/	1	/	/	/	/
Saremco	6	/	/	4	/	1	/	1
Schütz Dental	8	/	/	1	/	/	/	1
Septodont	7	/	/	1	/	/	/	/
Shofu	10	/	/	/	/	1	/	2
SDI	11	/	/	/	/	/	/	/
Sun Medical	3	/	/	/	/	/	/	1
Tokuyama	11	/	/	3	/	/	/	2
TP Orthodontics	/	/	1	/	1	/	/	/
Ultradent	3	1	2	3	/	2	/	4
Vericom	/	/	1	/	1	/	/	/
Voco	27	3	2	2	/	4	1	5
Total	266	29	47	81	19	24	10	67

**Table 2:** The different terms mentioned in MSDS when monomer composition was incomplete.

Terms Mentioned in MSDS (for Materials with Incomplete Compositions)	Number of Materials Concerned
Methacrylates	18
Hydrophobic aromatic dimethacrylate	10
Dimethacrylates	8
Uncured methacrylate ester monomers	7
Blend of multifunctional methacrylates	6
Hydrophobic aliphatic dimethacrylate	6
Acid adhesive monomer	6
Hydrophilic aliphatic methacrylate	6
Acidic monomer	6
Hydrophilic dimethacrylates	4
Acrylic monomers	4
Phosphoric acid monomer	4
Uncured acrylate ester monomers	4
Trade secret	3
Other	3
Phosphonic acid type monomer	3
Carboxilic acid type monomer	3
Hydrophilic amide monomer	3
Dimethacrylate cross linker	3
Copolymer of acrylic and itaconic acid	3
Aliphatic dimethacrylate	2
Uncured methacrylate resin mixture	2
Phosphatic methacrylate monomer	2
Mixture of uncured methacrylate ester monomers	2
Acidic and hydrophilic methacrylic monomers	2
Acrylates	2
Hydrophilic acidic monomer	2
Other bifunctional methacrylate monomers	1
Aromatic dimethacrylate	1
Aliphatic trimethacrylate	1
Matrix of methacrylic monomers	1
Methacrylate ester monomer	1
Polymerizable dimethacrylate resin	1
Polymerizable trimethacrylate resin	1
Citric acid methacrylate oligomer	1
Multifunctional monomers	1
Hydrophobic aromatic methacrylate	1
Proprietary methacrylate	1
Mixture of methacrylate monomers	1

**Table 3:** Monomers found in chemical compositions of all materials. List of their names and/or chemical names.

Monomer Abbreviation	Monomer Name and/or Chemical Name
BisGMA	Bisphenol A Glycidyl Methacrylate or 2,2-bis[4-(3-methacryloxy-2-hydroxypropoxy)phenyl]propane
PC BisGMA	Polycarbonate-modified bis-GMA
BisDMA	Bisphenol A Dimethacrylate or 2,2-bis-(4-(methacryloxy) phenyl) propane
BisEMA or EBPADMA or E2BADMA	Ethoxylated Bisphenol A glycol dimethacrylate
BisMPEPP or BPEDMA	Bisphenol A polyethoxy dimethacrylate or 2,2-bis(4-methacryloxy poly-ethoxyphenyl)propane
BisPMA	Propoxylated Bisphenol A-Dimethacrylate
BisGDMAP	Bis(Glyceryl Dimethacrylate) Phosphate or 2-methacryl acid phosphinicobis (oxy-2,1,3-propanetriyl) ester
UDMA/UDMA modified	Urethane dimethacrylate or 1,6-di(methacryloyloxyethylcarbamoyl)-3,3,5-trimethylhexane
UTMA	Urethane trimethacrylate
AUDMA	Aromatic urethane dimethacrylate
TEGDMA	Triethylene glycol dimethacrylate
TEDMA	Triethylene dimethacrylate
DEGDMA	Diethylene glycol dimethacrylate
EGDMA	Ethylene glycol dimethacrylate
PEGDMA	Polyethylene glycol dimethacrylate
PEGDA	Polyethylene glycol diacrylate
PMMA	Polymethyl methacrylate
MMA	Methyl methacrylate
HEMA	Hydroxyethyl methacrylate or -Propenoic acid, 2-methyl-, 2-hydroxyethyl ester or 2-hydroxyethyl methacrylate
HPMA	2-Hydroxypropyl methacrylate
GDMA	Glycerol dimethacrylate
GMA	Glycidyl methacrylate
GPDM	Gycerol phosphate dimethacrylate
DMAEMA	2-(Dimethylamino)ethyl methacrylate or Methacrylic acid 2-(dimethylamino)ethyl ester
BDDMA	1,4-Butanediol Dimethacrylate or Tetramethylene dimethacrylate
THFMA	Tetrahydrofurfuryl methacrylate or 2-Propenoic acid, 2-methyl-, (tetrahydro-2-furanyl)methyl ester
TMPTMA	Trimethylolpropane trimethacrylate
TMPTA	Triméthyllolpropane triacrylate or 2-propenoic acid, 2-ethyl-2-((1-oxo-2-propenyl)oxy)methyl)-1,3-propanediyl ester
TMPSM or TMSPMA	3-(Trimethoxysilyl)propyl methacrylate or 3-Methacryloxypropyltrimethoxysilane
HDODA	1,6-Hexanediol diacrylate
4-MET	4-methacryloxyethyl trimellitic acid
4-META	4-methacryloyloxyethy trimellitate anhydride
10-MDP	10-Methacryloyloxydecyl dihydrogen phosphate
MDTP	10-methacryloyloxydecyl dihydrogen thiophosphate
NPG2PODA	Neopentyl glycol propoxylate diacrylate
NPGDMA	Neopentyl glycol Dimethacrylate or 2,2-dimethylpropane-1,1-diyl bis(2-methylprop-2-enoate)
NTGGMA	N-(2-hydroxy-3-((2-methyl-1-oxo-2-propenyl) oxy) propyl)-N-tolyl glycine
TCDDMA or TCDMA	Tricyclodecane dimethanol dimethacrylate or 2-propenoic acid,(octahydro-4,7-methano-1h-indene-5,1-diyl)bis(methylene) ester
D3MA	decanediol 1,10-dimethacrylate
PCDMA	Polycarbonate dimethacrylate
TCD-DI-HEA	2-propenoic acid; (octahydro-4,7-methano-1H-indene-5-diyl) bis(methyleneiminocarbonyloxy-2,1-ethanediyl) ester
DDCDMA	Dimer dicarbamate dimethacrylate
LPS monomer	proprietary monomer
IBMA	Isobutyl methacrylate
PDMA	Polybutanediol dimethacrylate 600
PMGDM	Pyromellitic dianhydride glycerol dimethacrylate
AMPS	2-Acrylamido-2-methylpropane sulfonic acid ou 2-Acrylamido-2-methylpropane sulfonic acid
BMEP	Bis[2-(methacryloyloxy)ethyl] phosphate
PENTA	Dipentaerythritol penta-acrylate phosphate
MPTMS	3-Mercaptopropyl trimethoxysilane
PMDM	Pyromellitic dimethacrylate
TCDDA	Tricyclodecane dimethanol diacrylate or Tricyclo[5.2.1.02,6]decanedimethanol diacrylate
AHPM	3-(acryloyloxy)-2-hydroxypropyl methacrylate
PPTTA	ethoxylated (5.0) pentaerythritol tetraacrylate
AFM	Proprietary monomer
SDR	Proprietary monomer
DDDMA	1,12-Dodecanediol dimethacrylate or 12-(2-methylprop-2-enoyloxy)dodecyl 2-methylprop-2-enoate
HDMA or HDDMA or HEDMA	1,6 Hexanediol dimethacrylate
ETPTA	Trimethylolpropane ethoxylate triacrylate

**Table 4:** Monomer composition for all categories of resin-based materials included in the analysis.

Monomer Composition	Restorative Composites	Core Build-Up Composites	Orthodontic Composites	Restorative Adhesives	Orthodontic Adhesives	Sealants	Restorative RMGICs	Luting Cements and Composites	Total
With BPA derivatives	223 (83.8%)	24 (82.8%)	37 (78.7%)	36 (44.4%)	12 (63.2%)	16 (66.7%)	3 (30%)	31 (46.3%)	382 (70.3%)
With BisGMA	177 (66.5%)	20 (69%)	29 (61.7%)	31 (38.3%)	11 (57.9%)	12 (50%)	0	25 (37.3%)	305 (56.2%)
With BisEMA	109 (41%)	11 (37.9%)	7 (14.9%)	5 (6.2%)	2 (10.5%)	3 (12.5%)	3 (30%)	12 (17.9%)	152 (28%)
With BisDMA	1 (0.4%)	0	1 (2.1%)	2 (2.5%)	2 (10.5%)	2 (8.3%)	0	0	8 (1.5%)
With BisMPEPP	15 (5.6%)	0	0	1 (1.2%)	0	0	0	1 (1.5%)	17 (3.1%)
With BisPMA	3 (1.1%)	0	0	0	0	0	0	0	3 (0.6%)
With PC BisGMA	2 (0.8%)	0	0	0	0	0	0	0	2 (0.4%)
With UDMA	188 (70.7%)	14 (48.3%)	12 (25.5%)	27 (33.3%)	7 (36.8%)	15 (62.5%)	4 (40%)	42 (62.7%)	309 (56.9%)
With TEGDMA	175 (65.8%)	23 (79.3%)	20 (42.6%)	20 (24.7%)	6 (31.6%)	11 (45.8%)	1 (10%)	36 (53.7%)	292 (53.8%)
With HEMA	10 (3.8%)	0	6 (12.8%)	71 (87.7%)	10 (52.6%)	3 (12.5%)	9 (90%)	25 (37.3%)	134 (24.7%)
Without BPA derivatives	43 (16.2%)	5 (17.2%)	10 (21.3%)	45 (55.6%)	7 (36.8%)	8 (33.3%)	7 (70%)	36 (53.7%)	161 (29.7%)
Without BPA derivatives or UDMA, TEGDMA and HEMA	7 (2.6%)	0	3 (6,4%)	6 (7.4%)	1 (5.3%)	2 (8.3%)	1 (10%)	1 (1.5%)	21 (3.9%)
Total	266	29	47	81	19	24	10	67	543

## Data Availability

The data presented in this study are available on request from the corresponding author.

## Supplementary Materials

The following supporting information can be downloaded at https://www.mdpi.com/article/10.3390/polym16081022/s1: Table S1: manufacturers and number of materials first identified for each category.
