# Supplementary material for: Analysis of Resin-Based Dental Materials’ Composition Depending on Their Clinical Applications
Source: Polymers (Basel). 2024 Apr 9;16(8):1022. doi: 10.3390/polym16081022 (PMC11053636; doi:10.3390/polym16081022)
Supplement: Supplementary file 1 [file polymers-16-01022-s001.zip › polymers-2935607-supplementary.pdf]

Supplementary material: Table S1. manufacturers and number of materials first identified for each category

Manufacturer	Restorative composite resins	Core build up composite resins	Orthodontic composte resins
**Apol**	7	4	/
**American orthodontics**	/	/	4
**Bisico**	12	4	/
**Biodinamica**	/	/	1
**BJM**	/	/	2
**Cavex**	3	/	/
**Centrix dental**	3	5	/
**Coltene**	11	1	/
**Cosmedent**	8	1	/
**CyberTech**	2	1	/
**Dentaurum**	/	/	3
**DenMat**	4	1	/
**Dental Technologies**	5	2	2
**Dentsply**	14	2	/
**DMG**	6	2	/
**DMP**	/	/	1
**Elsodent**	5	3	/
**Exotec**	5	2	/
**FGM**	6	1	2
**GC**	20	1	3
**Gestenco**	/	/	1
**Henry Schein**	7	/	/
**Itena**	2	1	/
**Ivoclar-Vivadent**	15	2	1
**Jeneric Pentron**	5	2	/
**Kent Dental**	6	/	/
**Kerr**	11	2	/
**Kettenbach Dental**	2	1	/
**Kulzer**	15	/	/
**Kuraray**	13	3	/
**Leone**	/	/	2
**3M**	13	/	6
**Micerium**	5	/	/
**Ormco**	/	/	4
**Ortho Technology**	/	/	3
**Parkell**	3	2	/
**Prime Dental**	/	/	3
**Pulpdent**	2	1	1

**Reliance**	/	/	13
**RMO**	/	/	5
**R&S**	6	/	/
**Saremco**	6	/	/
**Schütz Dental**	8	/	/
**Septodont**	7	1	/
**Shofu**	10	/	1
**SDI**	11	/	/
**Sun Medical**	4	/	/
**Tokuyama**	11	/	/
**TP Orthodontics**	/	/	3
**Ultradent**	5	1	2
**Vericom**	/	/	1
**Voco**	27	3	2
**Total**	**305**	**49**	**66**

Restorative adhesive systems	Orthodontic adhesive systems	Sealants	Resin modified glass ionomer cements
3	/	1	/
/	3	/	/
9	/	1	/
/	/	/	/
/	1	/	/
2	/	/	/
1	/	/	/
5	/	/	/
1	/	2	/
2	/	1	/
/	1	/	/
6	/	1	2
4	1	2	/
5	/	1	/
4	/	/	/
/	/	/	/
4	/	1	/
4	/	1	/
2	/	/	/
5	/	/	3
/	/	/	/
3	/	1	1
2	/	1	/
8	/	4	/
2	/	/	1
2	/	/	/
5	/	/	/
/	/	/	/
3	/	/	/
7	/	1	/
/	1	/	/
8	3	2	5
3	/	1	/
/	1	/	/
/	1	/	/
3	/	/	/
/	2	/	/
3	1	2	1

/	10	/	/
/	2	/	/
2	/	/	/
5	/	1	/
2	/	/	/
2	/	/	/
4	1	1	/
3	/	1	2
1	/	/	/
6	/	/	/
/	3	/	/
5	/	2	/
/	1	/	/
6	1	4	1
**142**	**33**	**32**	**16**

Luting cements and composites

3

/

4

/

/

/

1

3

2

/

/

/

2

3

2

/

2

3

2

10

/

2

3

5

4

/

5

1

1

5

/

5

4

/

/

2

/

3

/
/
/
1
1
2
2
4
1
2
/
4
/
6
**100**
